# A novel and polarization insensitive triband absorber using a non-uniform design approach on a flexible material for X-band applications

**DOI:** 10.1371/journal.pone.0336457

**Published:** 2026-04-30

**Authors:** N. V. Satyanarayana, G. R. K. Prasad, Nagandla Prasad, Om Prakash Kumar, B. T. P. Madhav, Praveen Kumar

**Affiliations:** 1 Department of ECE, ALRC-R&D, Koneru Lakshmaiah Education Foundation, Vaddeswaram, Andhra Pradesh, India; 2 Department of Electronics and Communication Engineering, GMR Institute of Technology, Rajam, Andhra Pradesh, India; 3 Manipal Institute of Technology, Manipal Academy of Higher Education, Manipal, India; Universiti Brunei Darussalam, BRUNEI DARUSSALAM

## Abstract

To address the issue of satisfying absorption levels in the microwave X-band region, this study proposes a tri-band metasurface absorber that makes use of a new non-uniform technique. A unit cell is used to form the proposed absorber. This unit cell is printed on a jeans dielectric substrate, which has a dielectric constant of 2.2. copper material is chosen as a radiating patch for the proposed unit cell. The radiating patch contains different polygons with different sizes. The structure has been designed in four phases to have three absorption peaks. The structure that is being proposed is resonating at three different resonant frequencies, which are 9.98GHz, 10.43GHz, and 11.30GHz. Irrespective of polarization angles and mode of propagation, the structure produced absorptances as 95.1, 97.3, and 99.89, respectively for the corresponding resonant frequencies. The absorber that has been proposed possesses an electro-magnetic characteristic that is effective for microwave applications, particularly for satellite and radar telecommunications. Additionally, bending analysis confirms the robustness of the design under conformal conditions, making it suitable for practical X-band applications such as radar cross-section reduction, satellite communication systems and electromagnetic interference mitigation. This work contributes to SDG 9: United Nations Sustainable Development Goal 9 by advancing innovative, flexible electromagnetic absorber design for next-generation X-band applications.

## 1. Introduction

A metasurface is made up of a collection of unit cells that can assist in the stimulation of surface waves. Due to their prospective benefits in comparison to conventionally available materials and technologies, metamaterials have garnered considerable attention. This allows their use in a wide range of devices, such as polarizers, detectors, and absorbers, over many different frequency regimes (microwave, terahertz, ultraviolet, visible, infrared…etc). A metasurface layer is often composed of numerous periodic unit cells that can have a variety of shapes, including squares, rectangles, H shapes, and more. Improving antenna performance through the use of metasurfaces has recently become more important. As was reported in [1], metasurface can be utilized for the purpose of enhancing antenna gain and bandwidth improvement [2]. Adding a metasurface above [3] or below the radiating element [4] is a great way to improve the antenna's performance matrices. This method garnered significant attention because it enhanced antenna performance by generating additional resonances through the surface wave excitation of a radiating patch, a novel concept. Non-uniformity is another method for enhancing the antenna's effectiveness. Many different types of metasurfaces, including focusing lenses, take advantage of nonuniform geometries and designs [5,6]. As stated in [7], a nonuniform metasurface is constructed to increase antenna gain. According to [8], a nonuniform metasurface can achieve a wide bandwidth. Landy et al. published the initial MA in 2008 [9].

There have been several proposals for metamaterial absorber designs covering various frequency bands. In [10], the authors suggest a polarization-independent MA with broadband absorption capabilities. However, the results reveal that at 8.5 and 15.5 GHz frequencies, the obtained peak absorption is 72.0% and 91.0%, respectively, which ultimately indicates low absorption. According to Chen et al., they created a double-circle-ring-based broadband absorption enriched MA that works with frequencies up to 7.5 GHz and doesn't depend on polarization and achieved more than 90% absorption [11]. Moreover, an electric SRR patch-based wideband absorber was presented by Zhao et al. [12], that offers a reflection coefficient of less than 10 dB from 4.40 GHz and 18.0 GHz. Various models are analysed by the researchers on absorbers, metasurfaces with uniform and few non uniform structures [13–25].

A small-sized V-shaped metamaterial absorber that works in X- and Ku-bands. The device has high efficiency electromagnetic absorption and polarization free over a wide incidence angle span. Amazingly, two resonant frequencies produce a near-total absorption, all on an electrically small footprint, and thus outperforming most prior designs. This design has competitive advantages around energy harvesting as well as in communication systems, defence and stealth technology [26]. A dual-band absorber designed on FR4 substrate. It demonstrates absorption over 98% at 1.8 GHz and 3.5 GHz with both angular and polarization stability. This design uses altered circular ring resonators, the behaviour of which is strictly checked by equivalent-circuit modelling, electromagnetic field modelling and careful experimental measurements. The fact that the simulation data and the empirical results adopted is close proves the effectiveness of the absorber in shielding microwave especially in GSM (1.8GHz) and sub-6 GHz 5G frequencies [27]. A simple multiband absorber constructed of asymmetric copper ring resonators on an FR4 substrate, which is designed to work in the microwave range. This architecture achieves five different absorption bands each with high efficiency and strong performance over a wide range of incident angles as is supported by CST simulations [28].

A small five-band metamaterial absorber that makes use of a star-shaped split-ring resonator, confined in a square shape, is fabricated on the cost-effective FR-4 substrate to be used in microwave applications. The design demonstrates high absorption of the S, C, X, and Ku bands [29]. five band metamaterial absorbers of a butterfly shaped elliptical ring resonator which is working in the terahertz domain (0.1–3 THz). The resonator can achieve unprecedented absorption at five different frequencies, with polarization insensitivity and strong performance even in the case of oblique incidence, hence its application across terahertz imaging, energy harvesting and sensing schemes [30]. A multiband metamaterial absorber which consists of two square split-ring resonators which have been optimized through genetic algorithm to be used at microwave frequencies. The design provides six high-efficiency absorption peaks in the Ku band, facilitated by the natural metamaterial resonance and zero-reflection effects, therefore, making it suitable in radar, energy-harvesting and sensing systems [31].

After reviewing the literature, the researchers of this study were motivated to create a new type of metasurface specifically for usage in the X-band. By considering the above literature we have propose a novel metasurface for X-band applications. The main salient features of the proposed work are as follows.

- The structure is a straightforward one and designed using three different layers
- Tribands are produced within the X-frequency band
- The structure is designed on a compact size material like polymide
- The bending analysis is performed for the structure and produced triband with a smaller difference
- E, H and current distributions are verified for the proposed design
- An equivalent circuit model is provided for the proposed design
- Parametric analysis is done for the proposed design’s radiating patch.

Although several tri-band and polarization-insensitive metamaterial absorbers have been reported in the literature, most existing designs rely on uniform resonator geometries, rigid substrates, or operate across widely separated frequency bands. Furthermore, limited attention has been given to achieving closely spaced multi-band absorption within the X-band using flexible substrates while maintaining angular and polarization stability. In this context, the novelty of the present work lies in:

- Introducing a non-uniform polygonal metasurface design strategy to generate multiple resonant modes within a compact unit cell.
- Achieving closely spaced tri-band absorption exclusively within the X-band, which is highly relevant for radar and satellite applications.
- Employing a flexible textile-based substrate, enabling conformal and wearable electromagnetic absorber implementations.
- Providing comprehensive validation through equivalent circuit modeling, angular stability analysis, polarization insensitivity, and bending performance.

The X-band frequency range (8–12 GHz) plays a critical role in modern radar, satellite communication, and defense systems. The proposed absorber’s tri-band operation within this range makes it particularly suitable for radar cross-section (RCS) reduction in stealth platforms, where selective absorption of radar signals is essential. Additionally, its polarization-insensitive and angularly stable behavior ensures consistent performance against arbitrary incident wave orientations, which is crucial in real-world radar scenarios.

## 2. Unitcell design process

**Fig 1:**
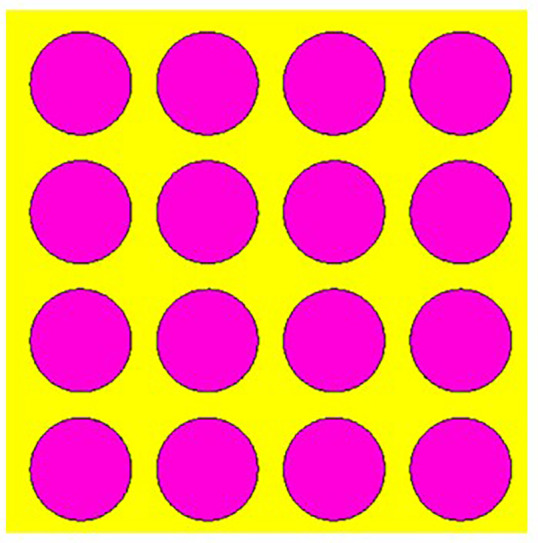
Step-1 of proposed unit cell.

**Fig 2:**
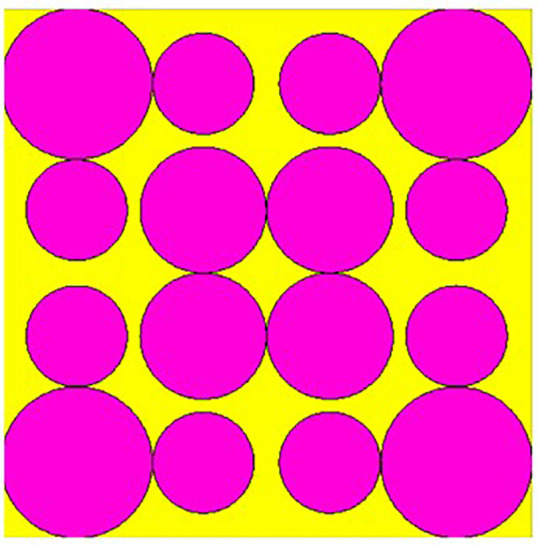
Step-2 of proposed unit cell.

**Fig 3:**
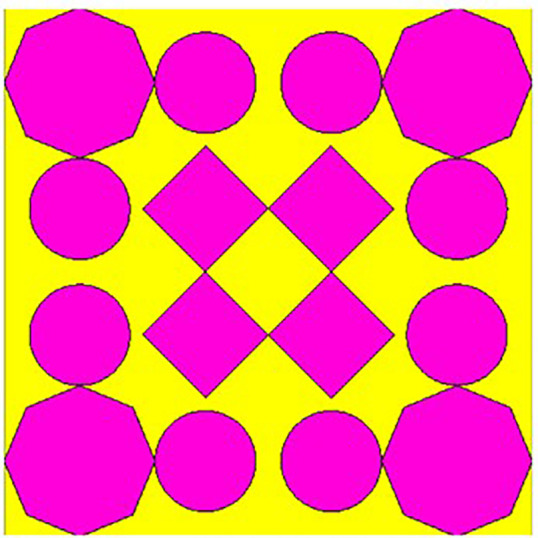
Step-3 of proposed unit cell.

**Fig 4:**
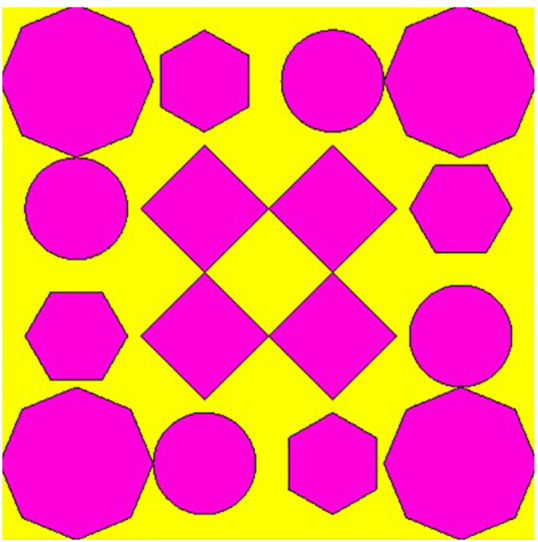
Step-4 of proposed unit cell.

In contrast to the conventionally available designs, non-uniformity is a novel approach used to improve the performance of an antenna or a metasurface. The proposed unit cell consists of three distinct layers. The proposed structure uses copper, a highly conductive material with a conductivity of 5.8e + 07 s/m and a thickness of 0.035 mm, for both the bottom and radiating patch layers. The bottom layer of the present design acts as a metal reflector. We have chosen polymide, with a thickness of 2 mm and a dielectric constant of 2.2, as the dielectric material for the proposed design. The magenta color indicates a radiating patch for the proposed design, while the yellow color indicates polymide. The proposed unit cell structure's overall dimension is 42 x 42 x 0.1 mm^3^. There are four iterative processes involved in the design process of the proposed unit cell, which are depicted as shown in Figs 1–4.

**Fig 5:**
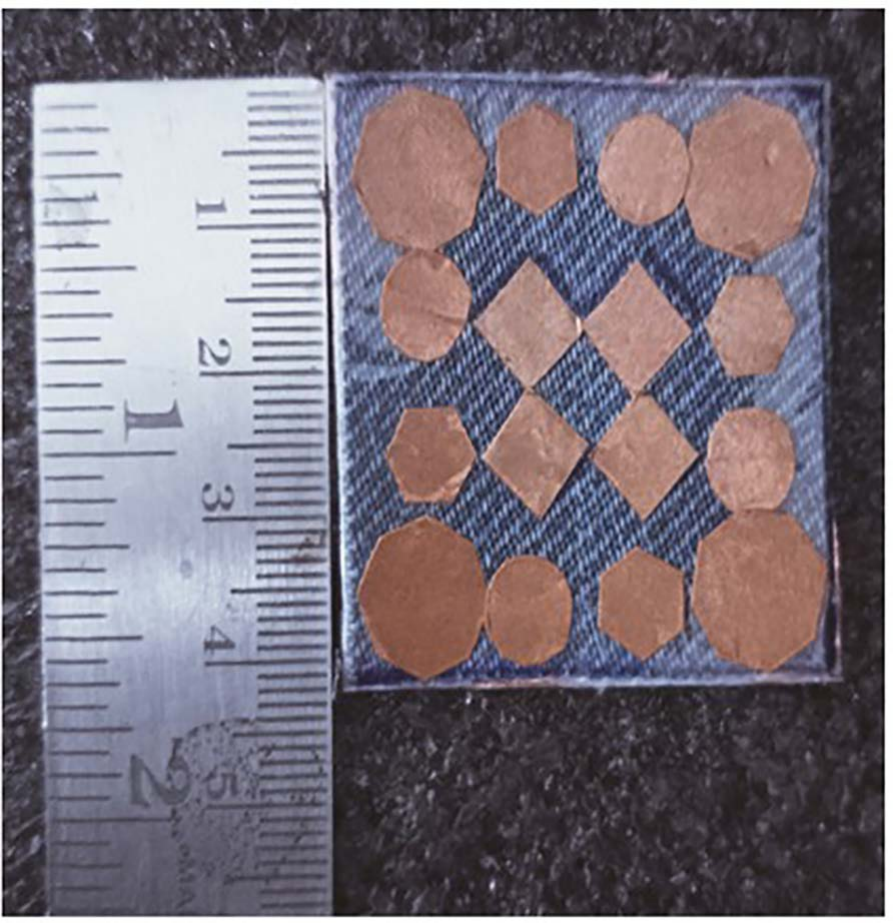
Top view of prototyped model.

**Fig 6:**
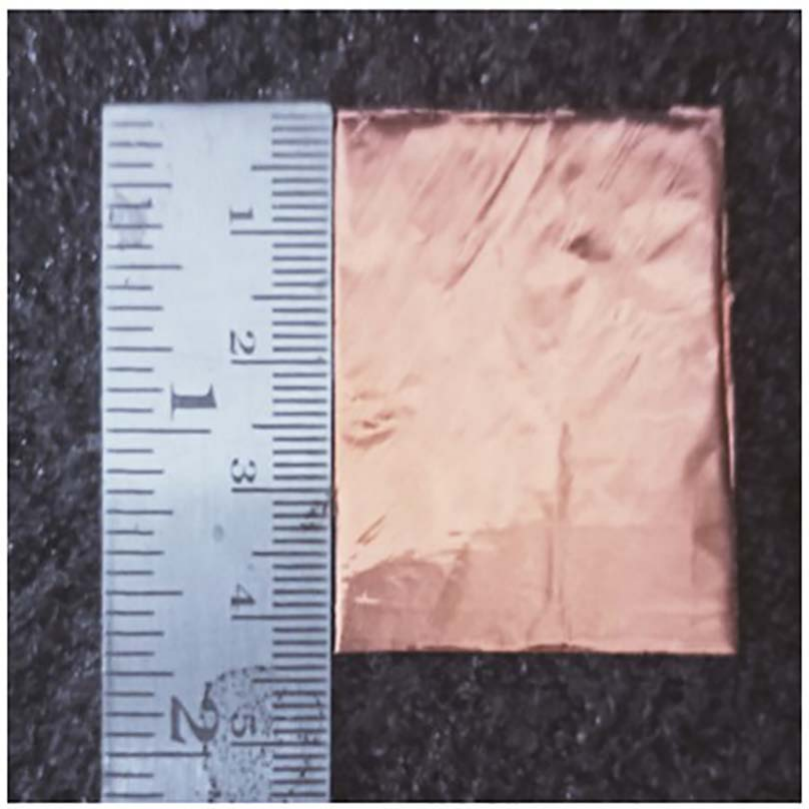
Bottom view of prototyped model.

**Fig 7:**
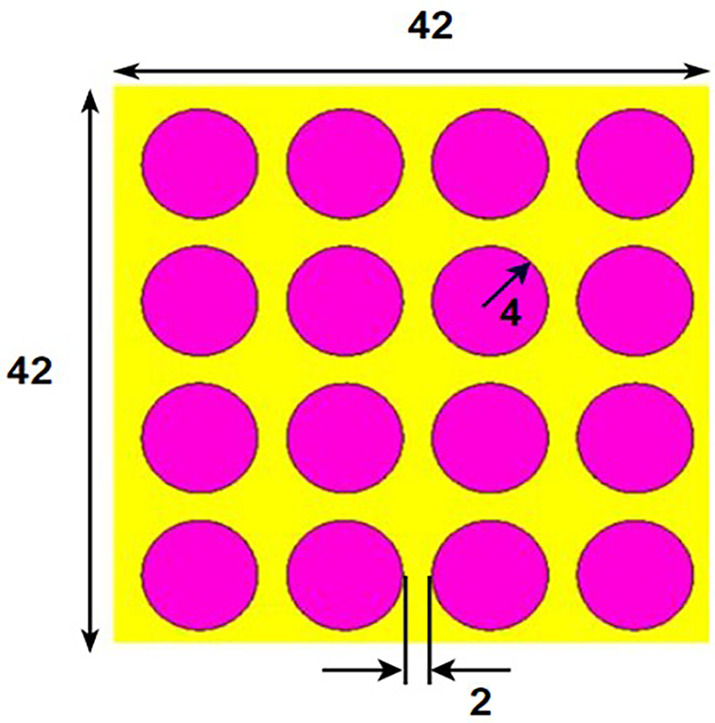
Dimensions of uniform circular rings.

**Fig 8:**
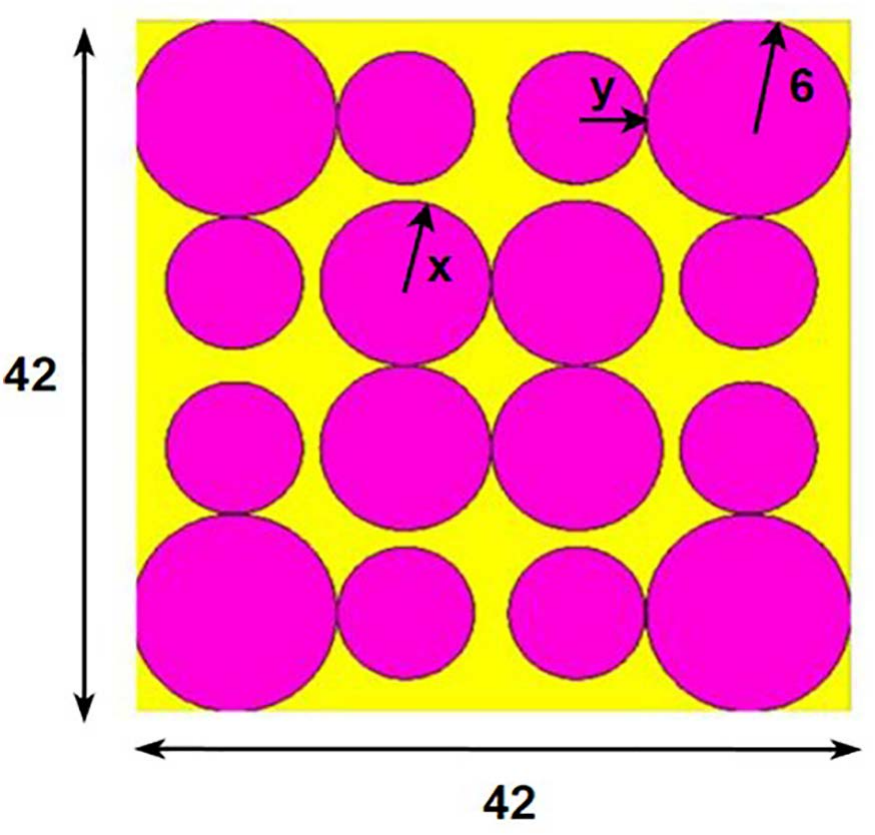
Dimensions of non-uniform circular rings.

**Fig 9:**
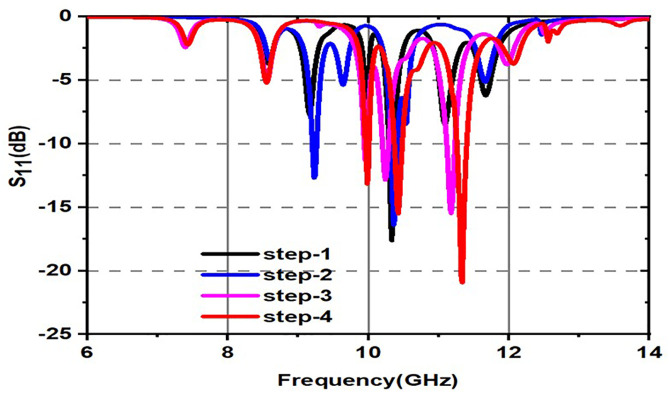
S_11_(dB) of the proposed unitcell for different steps.

As indicated in the Fig 1, the first step in designing the patch is to create an array with 4 × 4 sized circles and each circle consists of a radius of 4 mm and there is a 2 mm gap between any two circles. The next step involves altering the outermost circles of the radiating patch to a radius of 6 mm, as depicted in Fig 2. Immediately, the circles that are located at the center of the radiating patch are transformed into square shapes, and the circles that are located at the outermost corners of the patch are transformed into octagonal shapes, as shown in Fig 3, and finally, the shape of the radiating patch that has been proposed is depicted in Fig 4. Fig 5 and 6 show fabricated prototype and Figs 7 and 8 depict the dimensions of the proposed unit cell and Fig 9 illustrates the obtained S_11_(dB) response for various steps presented in Fig 9. The structure is finalized as shown in Fig 4 and it is producing three resonant frequencies as shown in Fig 9. The red-colored spectrum shown in Fig 9 indicates obtained S_11_ response for Fig 4. The finalized structure is resonating at three resonant frequencies of 9.98GHz, 10.43GHz and 11.30 GHz with S_11_ magnitudes of −13.76dB, −15.91dB and −21.59 dB respectively.

### 2.1. Simulation setup and absorptivity phenomenon

**Fig 10:**
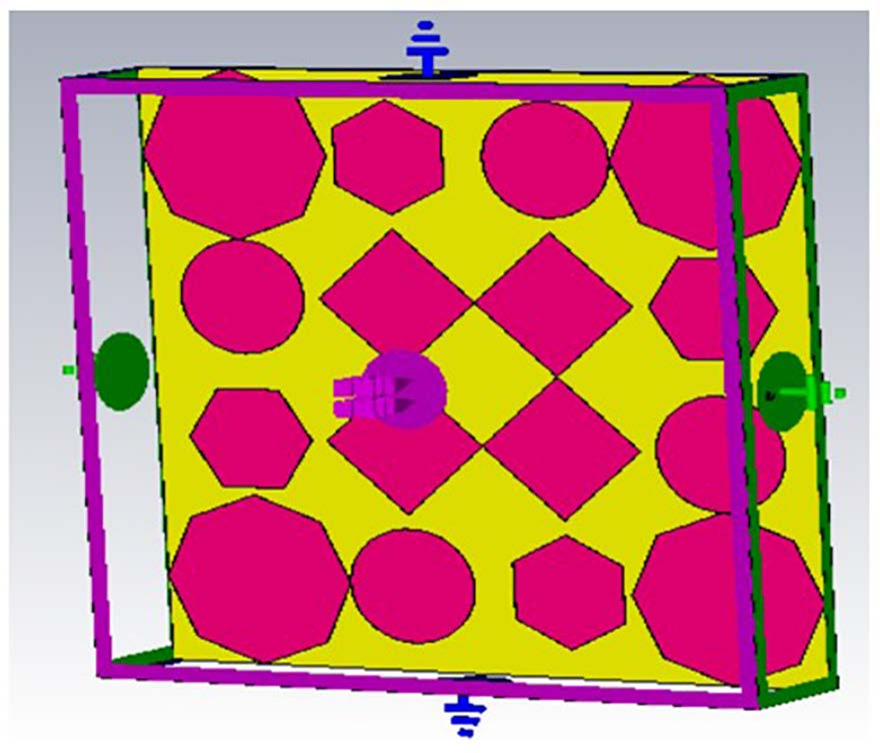
Simulation setup of the proposed unitcell in CST studio.

The proposed structure is implemented using CSTMW tool for performing numerical calculations. Using this tool, large radiating structures and even arrays with hundreds of radiating elements can be handled. The analysis of a unit cell array-based structures was numerically performed in CST microwave studio software using a frequency-domain (FD) solver. The simulation process initiates by choosing frequency limits (upper and lower) with a step size and immediately electric and magnetic boundary conditions are applied along X- and Y-directions respectively to impinge an EM wave along the Z-direction as shown in Fig 10. A plane wave excitation is applied along the Z-direction using Floquet ports.

**Fig 11:**
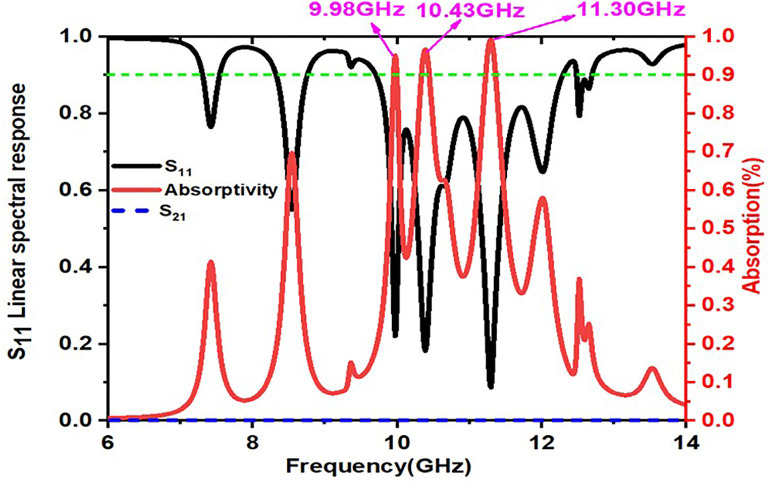
Absorption,transmission and reflection coefficients linear spectrum response of the proposed unitcell design.


The obtained absorptivity response of the proposed design is represented with red colored spectrum as shown in Fig 11. The following numerical equations are considered for calculating absorptivity response of a unitcell

$$\Gamma =\frac{Z-1}{Z+1}$$ (1)

$$Z=\sqrt{\frac{\mu_{r}}{\varepsilon_{r}}}$$ (2)

$$S_{11}=\frac{(1-\Gamma^{\text{2}})Z}{1-\Gamma^{\text{2}}Z^{\text{2}}}$$ (3)

Where Г, Z, and S_11_ are reflection coefficient, normalized impedance and return loss. Using the following formula, we can calculate the absorption spectrum for any unit cell.


$$\text{Absorption\textbackslash{}hspace\{0.33em}\}A=1-abs\left({S_{11}}^{\text{2}}\right)$$ (4)

The absorption, reflection and transmissions of the proposed unit cell is shown in Fig 4 From the Fig 11, we can notice that red coloured spectrum indicates absorption, black coloured spectrum indicates reflection(S_11_) and finally magenta coloured spectrum indicates transmission (S_21_) of the structure. Here, it is a fact that the proposed design is resonating at three resonant frequencies: 9.98GHz, 10.43GHz, and 11.30 GHz, with percent levels of absorption of 95.1, 97.3, and 99.89, respectively.

### 2.2. Metamaterial nature of the design

The effective dielectric medium parameters are crucial for a unit cell since they allow the manipulation of electromagnetic energy in a manner that is not possible with natural materials. This material may have a refractive index, epsilon, and mu that are all negative. The structure design and the nature of repetition primarily determine these distinctive characteristics.

**Fig 12:**
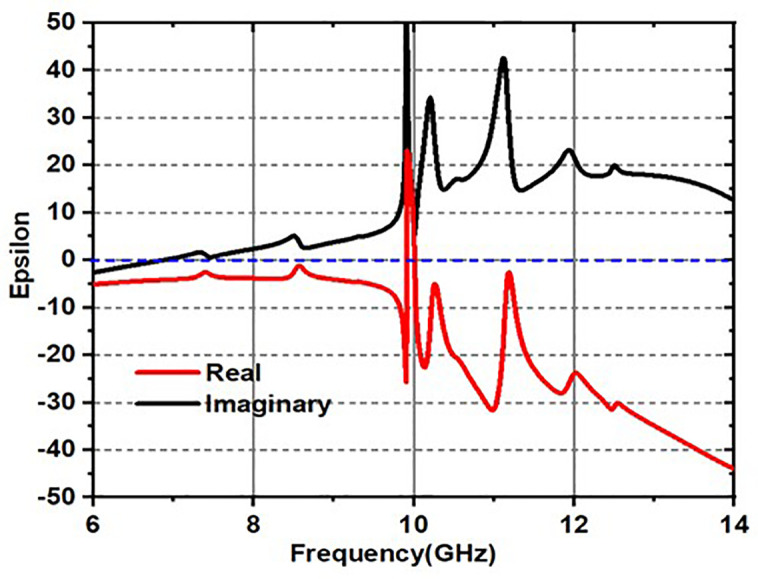
Epsilon response.

**Fig 13:**
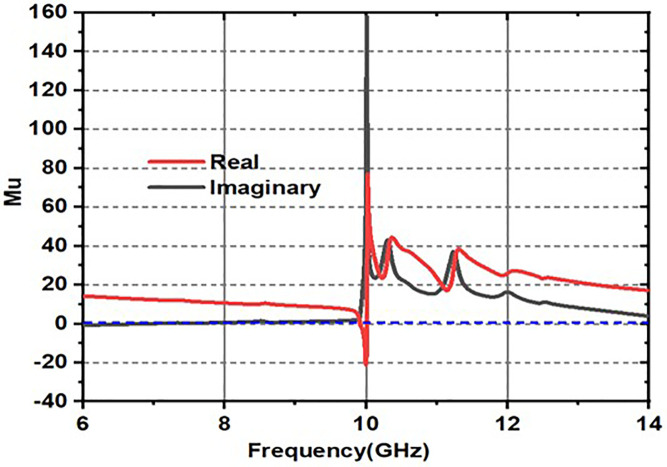
mu response.

**Fig 14:**
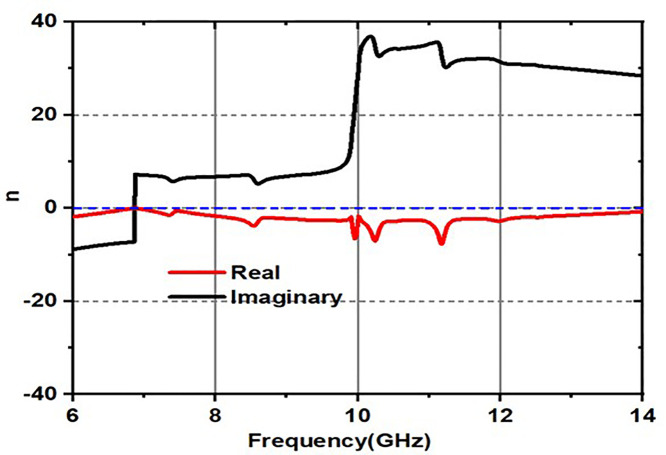
Refractive index response.

The structure is obviously showing metamaterial properties within the frequency of operation range, which can be clearly seen in Fig 12–14.

## 3. Result analysis

The proposed structure likely involves a layered material where EMwave undergoes multiple reflections at the interfaces between different layers. As wave reflects multiple times within these layers, each reflection can interfere with the others. Destructive interference occurs when the reflected waves are out of phase, leading to a reduction or cancellation of the light's intensity at certain wavelengths. This effect is highly dependent on the thickness of the layers, the wavelength of the light, and the refractive indices of the materials involved. The precise control of these parameters can be used to engineer specific optical properties, such as anti-reflective coatings or filters that selectively block certain wavelengths. The overall optical behavior of the structure is a result of the interplay between multiple reflections and destructive interference, which can be harnessed for various applications in optics and photonics. The enhanced tri-band absorption observed in the proposed metasurface originates from the deliberate introduction of geometric non-uniformity within the unit cell. Unlike uniform resonator arrays that support a single dominant LC resonance, the proposed design incorporates multiple polygonal elements of varying sizes and shapes, each supporting distinct surface current distributions and electrical path lengths.

**Fig 15:**
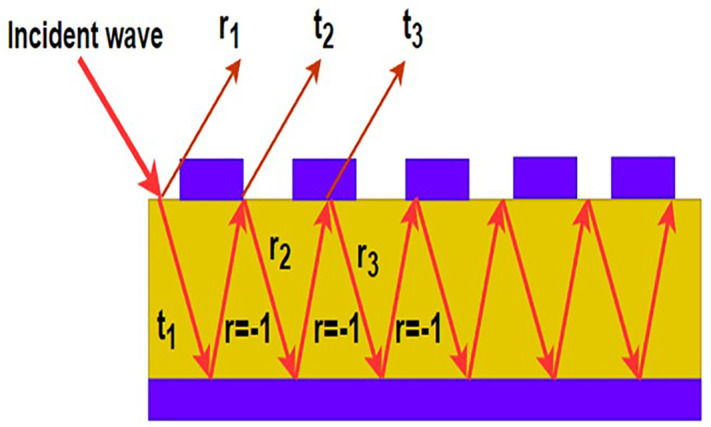
Representation of multiple refections and destructive interference theory of the proposed structure.

**Fig 16:**
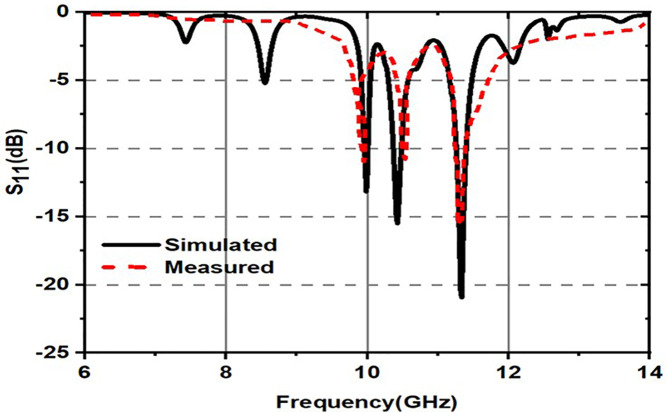
Simulated and measured s_11_(dB) of the proposed metasurface.

Fig 15 illustrates a structure where an incident wave undergoes multiple reflections and transmissions through a layered material. The incident wave enters the structure, where part of it is reflected back at each interface (denoted by r1,r2,r3) and part is transmitted through the layers (denoted by t1,t2,t3). Inside the structure, the wave undergoes multiple reflections between the layers, with each reflection possibly undergoing a phase shift, indicated by r=−1, signifying a phase change of 180 degrees (π radians). This phase change is crucial for destructive interference, where the reflected waves can cancel each other out if they are out of phase. The precise thickness and refractive index of the layers dictate the constructive or destructive interference pattern, influencing the overall transmission and reflection properties of the structure. This principle is commonly applied in designing optical coatings, filters, and other photonic devices. Fig 16 shows simulated and measured S11(dB) of the proposed metasurface.

**Fig 17:**
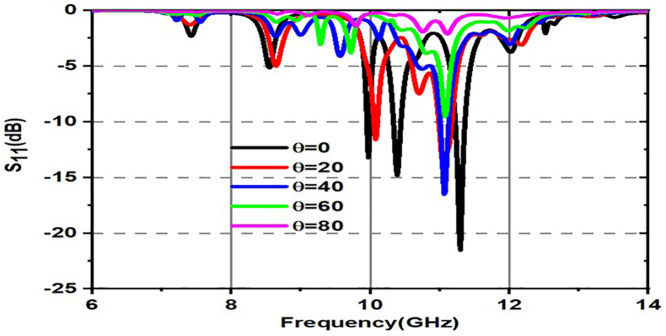
Variation of S_11_(dB) for various incident angles under TE mode.

**Fig 18:**
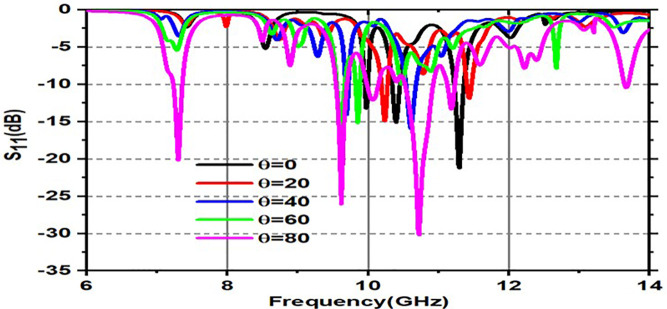
Variation of S_11_(dB) for various incident angles under TM mode.

Variation of S11(dB) for various incident angles for both TE and TM modes is shown in Fig 17 and 18. Fig 17 shows various incident angles under TEmode, and Fig 18 shows the frequency and angles of incidence S11 response.

**Fig 19:**
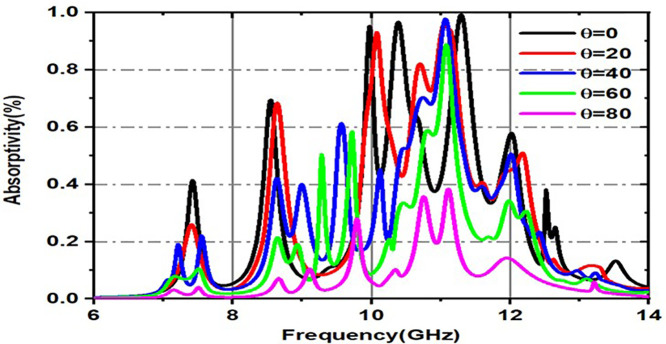
Variation of Absorptivity for various incident angles under TE mode.

**Fig 20:**
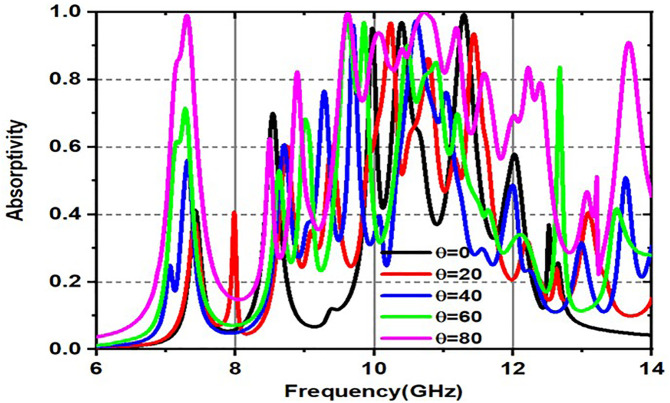
Variation of Absorptivity for various incident angles under TM mode.

Fig 19 and 20 are made up of two graphs that show how the absorptivity changes (in percent) over a frequency range (GHz) for different incident angles, especially for both TE (transverse electric) and TM (transverse magnetic). These graphs provide a visual representation of the relationship between absorptivity and frequency, specifically for incident angles (θ). The material absorbs more energy at corresponding resonant frequencies, which are represented by the peaks in the curves, which exhibit differing absorption levels for various incident angles. Fig 19 illustrates the fluctuation of absorptivity under TE mode across various frequencies and incidence angles, and the variation in absorption for various incident angles in TM mode is illustrated in Fig 20.

**Fig 21:**
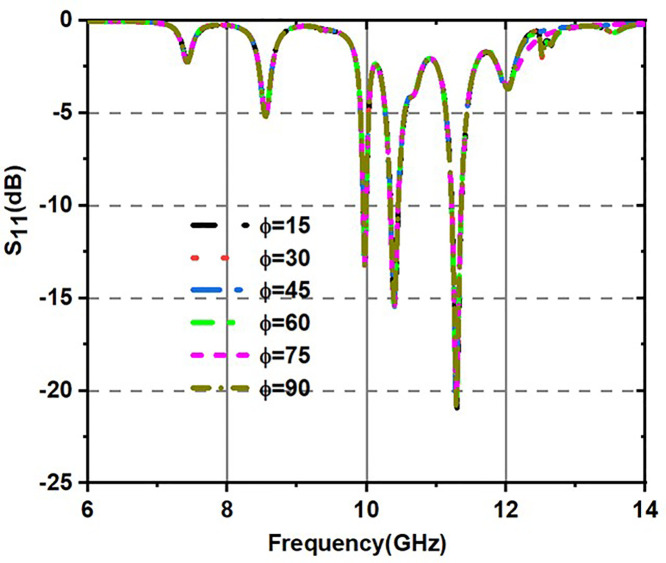
Variation of S_11_(dB) for various polarization angles under TE mode.

**Fig 22:**
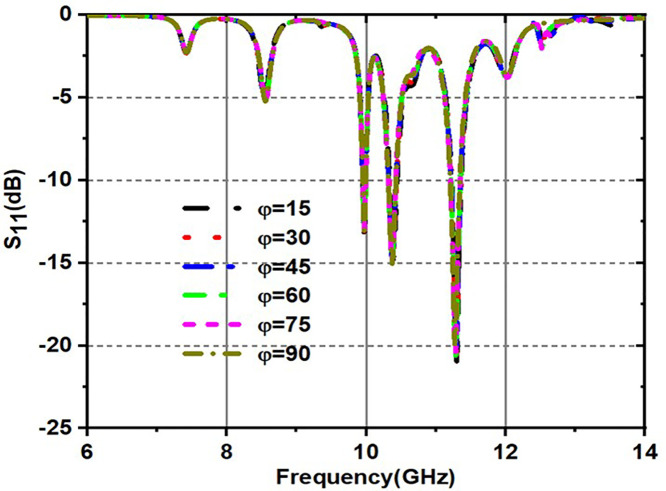
Variation of S_11_(dB) for various polarization angles under TM mode.

**Fig 23:**
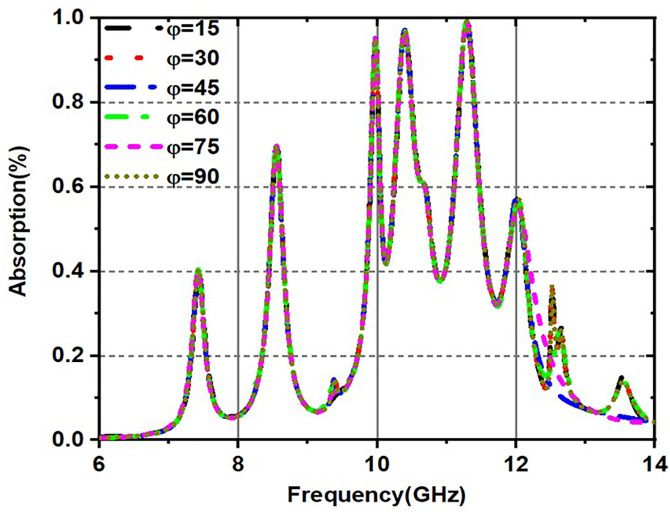
Variation of Absorptivity for various polarization angles under TE mode.

**Fig 24:**
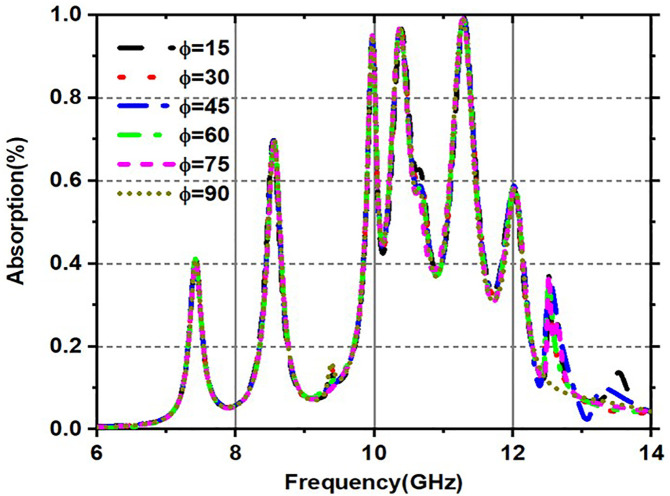
Variation of Absorptivity for various polarization angles under TM mode.

Fig 21 and 22 show the variation of S11(dB) for various polarization angles under TE and TM modes. Fig 23 and 24 show the variation of Absorptivity for various polarization angles under (a) TE and (b)TM modes

**Fig 25:**
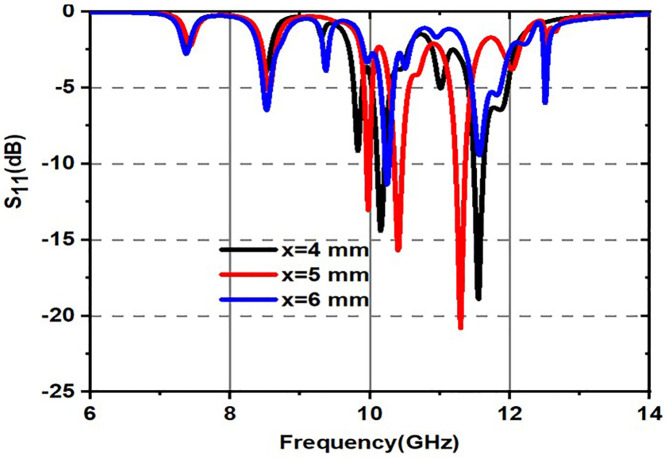
Variation of S_11_(dB) for radius of inner circles mentioned in the Figure.5.

**Fig 26:**
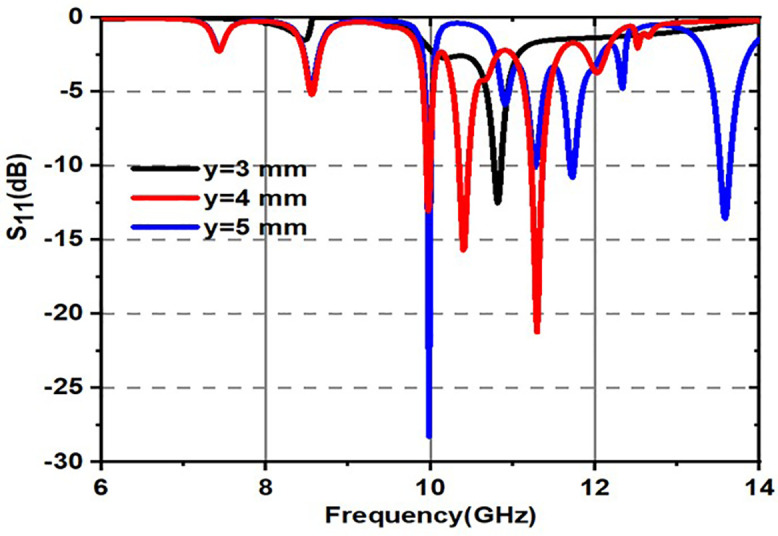
Variation of S_11_(dB) for outer most circles mentioned in the Figure.5.

Variation of S_11_ with inner circle radius is shown in Fig 25. From Fig 25, it is a fact that black colored spectrum indicates for x = 4 mm, red colored spectrum indicates for x = 5 mm and blue colored spectrum indicates for x = 5 mm. The x-axis represents frequency in the range of 6 GHz to 14 GHz, and the y-axis represents S_11_ in dB, which is ranging from 0 dB to −25 dB. As the radius of the inner circle increases from 4 mm to 6 mm, the positions of the resonances (sharp dips S_11_) shift slightly to lower frequencies.The minimum S_11_ value can be observed particularly at around 10 GHz.Variation of S_11_ with outer circle radius is shown in Fig 26. From Fig 26, it can be observed that there is a resonance shift in frequency as the radius of the outer circle increases and a better S_11_ magnitude can be obtained for obtained s11 corresponding to y = 4 mm.

### 3.1. Equivalent circuit model (ECM) analysis

**Fig 27:**
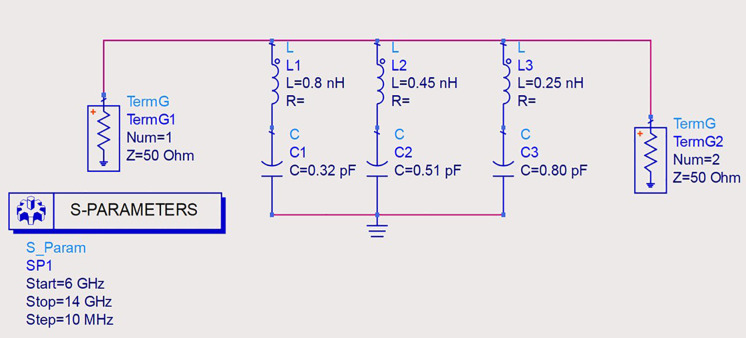
Equivalent circuit diagram for the proposed design.

**Fig 28:**
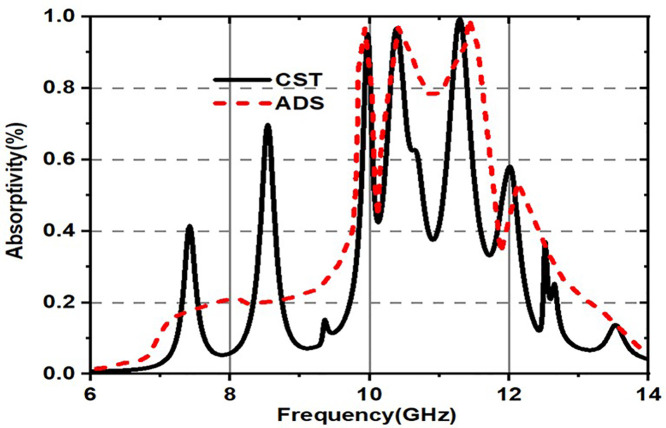
Absorption spectrum for the proposed unitcell using various tools.

The proposed metasurface’s ADS equivalent circuit design is demonstrated in Fig 27. From Fig 27, it is a fact that the outer octagonal shaped design with circle combination of the unitcell can be approximated as an inductor (L1) and the gaps between them can be modelled as capacitors(C1). Similarly, the squares present on the unitcell design can be modelled as as inductor (L2) and the gap present between them can be modelled as capacitors(C2) and finally hexagonal shaped patch can be modelled as an inductor (L3) and the gap present between them can be modelled as capacitors(C3). The dielectric material of the unitcell structure is set to 50-ohm impedance (Z). While doing the simulation the start and end frequencies set at 6 THz and 10 THz with a step size of 100 GHz. The obtained absorption spectrum is shown in Fig 28. Table 1 compares the proposed research.

**Table 1:** Comparision of the proposed antenna with existing antenna in the literature.

Ref	Unitcell size	Shape of the structure	Dielectric material used	Absorption peak frequencies	Absorptivity (%)	Polarization insensitive Nature	Flexibility
	**(mm**^**3**^)
[17]	14 × 14 × 1.6	Circular slot type	FR4	2.9	95	yes	no
				4.18	95.45		
				9.25	97.2		
[18]	10 × 10 × 1.6	Circular ring type	FR4	5.57	96.87	yes	no
				7.97	95.99		
				13.44	98.28		
[19]	13.8 × 13.8 × 1.6	Circular ring with an inner Jerusalem cross	FR4	4.4	93.13	yes	no
				6.05	96.41		
				13.9	96.36		
[20]	28.2 × 28.2 × 1.6	Square type ring with a Jerusalem cross	FR4	4.20	91	yes	no
				7.00	97.9		
				7.4	98.5		
[21]	12 × 12 × 1.6	Dumbbell	FR4	3.26	93.8	yes	no
				11.6	96.74		
				17.13	99.95		
[22]	35 × 35 × 1.6	Square and circular type SRR	Rogers	6.46	97.1	no	no
				7.68	91.2		
[23]	11.5 × 11.5 × 1.6	Jerusalem cross	PET	6.1	95	yes	yes
				9.2	96		
				11.6	97		
[24]	10 × 10 × 1.6	Rhombus	FR4_Epoxy	5.86	86.23	yes	no
				6.57	92.92		
				8.94	82.07		
[25]	10 × 10 × 0.1	Cross	Polymide	11.4	97.5	yes	yes
				19.2	96.5		
Current work	42 × 42 × 0.1	Non uniform size of different polygon shapes	Jeans	9.98	95.1	yes	yes
				10.43	97.3		
				11.3	99.89		

### 3.2. Bending analogy of the proposed metasurface

**Fig 29:**
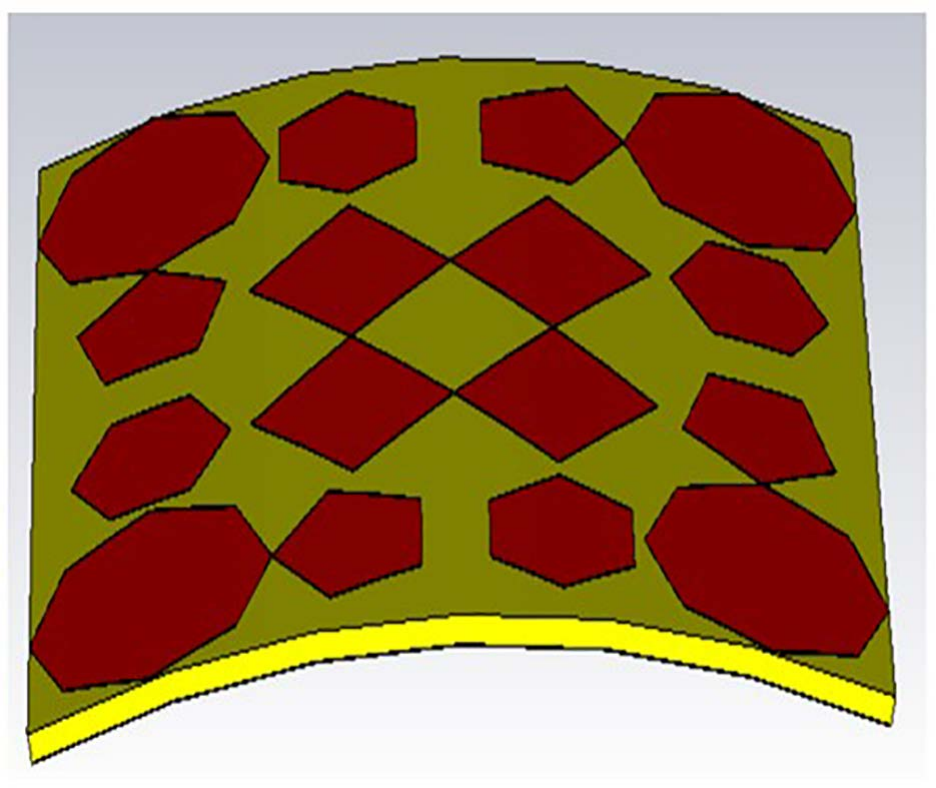
Bending position for 30 degrees.

**Fig 30:**
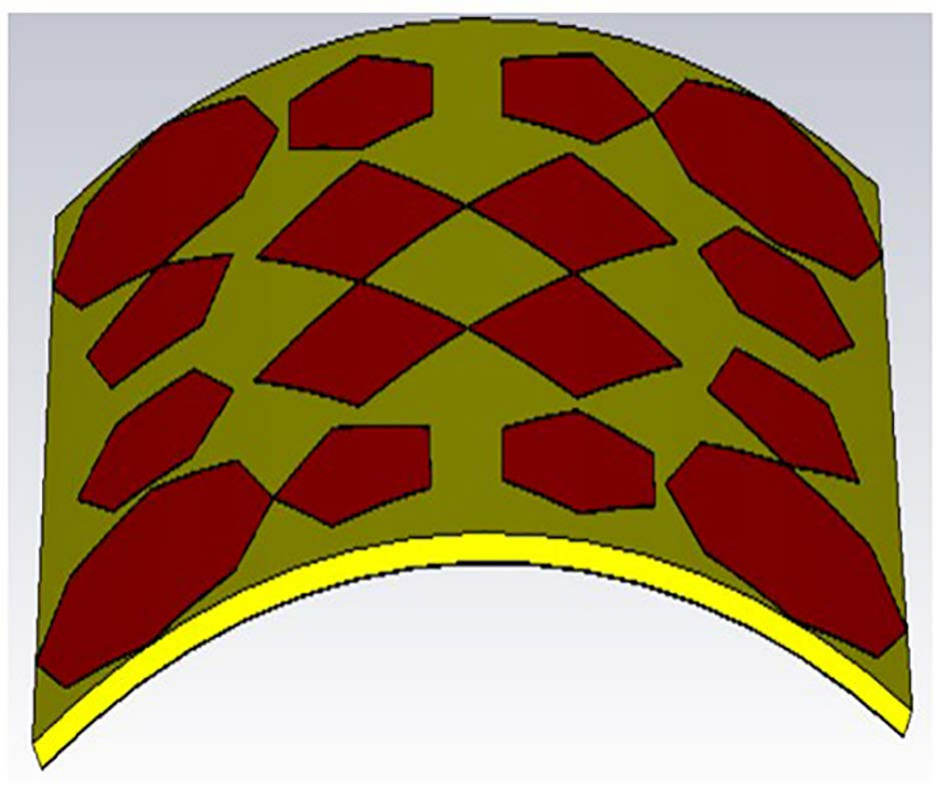
Bending position for 30 degrees.

**Fig 31:**
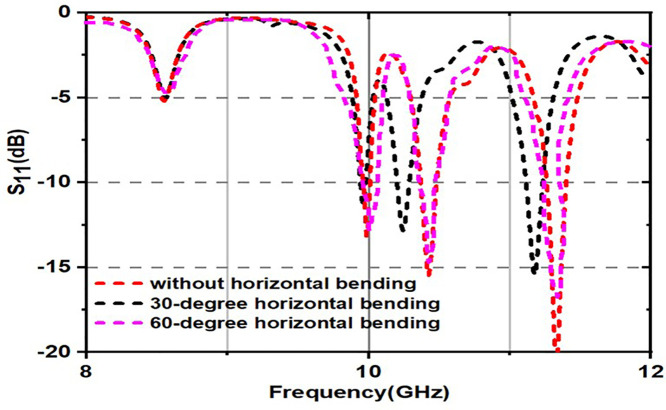
S_11_ characteristics for bending positions of the proposed unitcell.

As can be observed in Fig 29 and 30, with bending angles (30 and 60 degrees), the obtained S_11_ (dB) for both bending angles are almost the same; a good agreement between these results is obtained. It can also be noticed that there is too small a shift in resonant frequencies, as shown in the Fig 31. Although the proposed absorber operates based on multi-scale LC resonances, the bending analysis demonstrates negligible variation in resonance frequencies. This behavior can be attributed to the preservation of local resonant geometries under moderate bending conditions. The applied bending angles (30° and 60°) correspond to large bending radii, ensuring that inter-element spacing and local current loops remain largely unaffected.

### 3.3. Field distribution analysis

**Fig 32:**
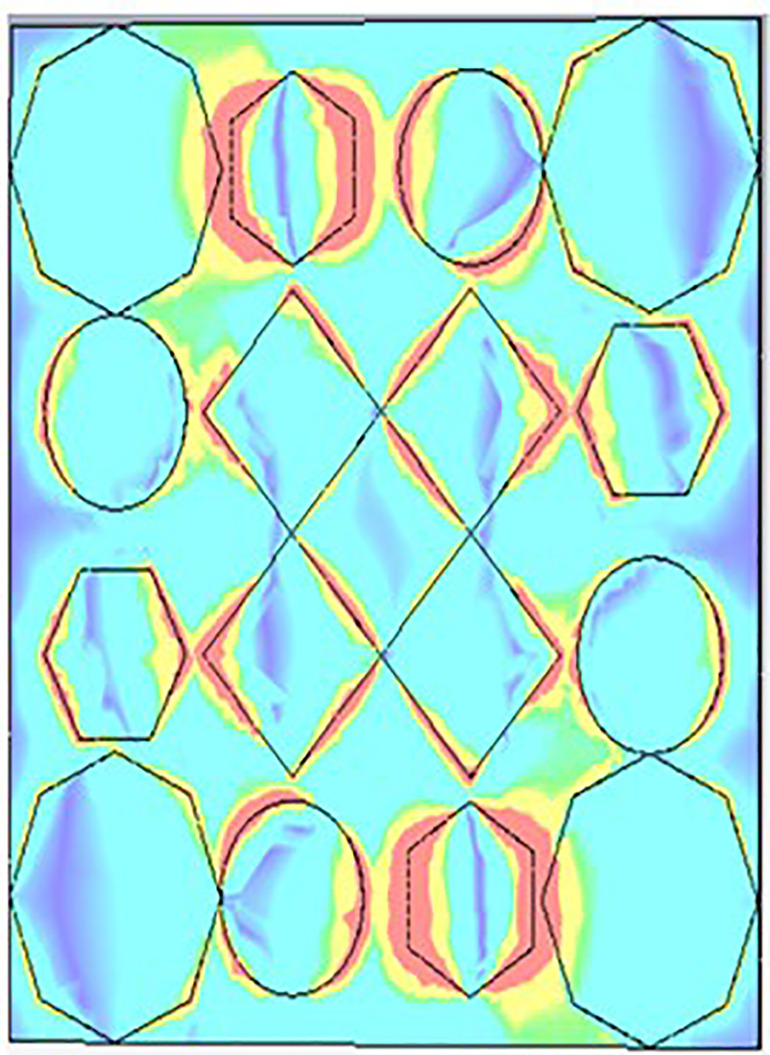
E- field at 9.93GHz.

**Fig 33:**
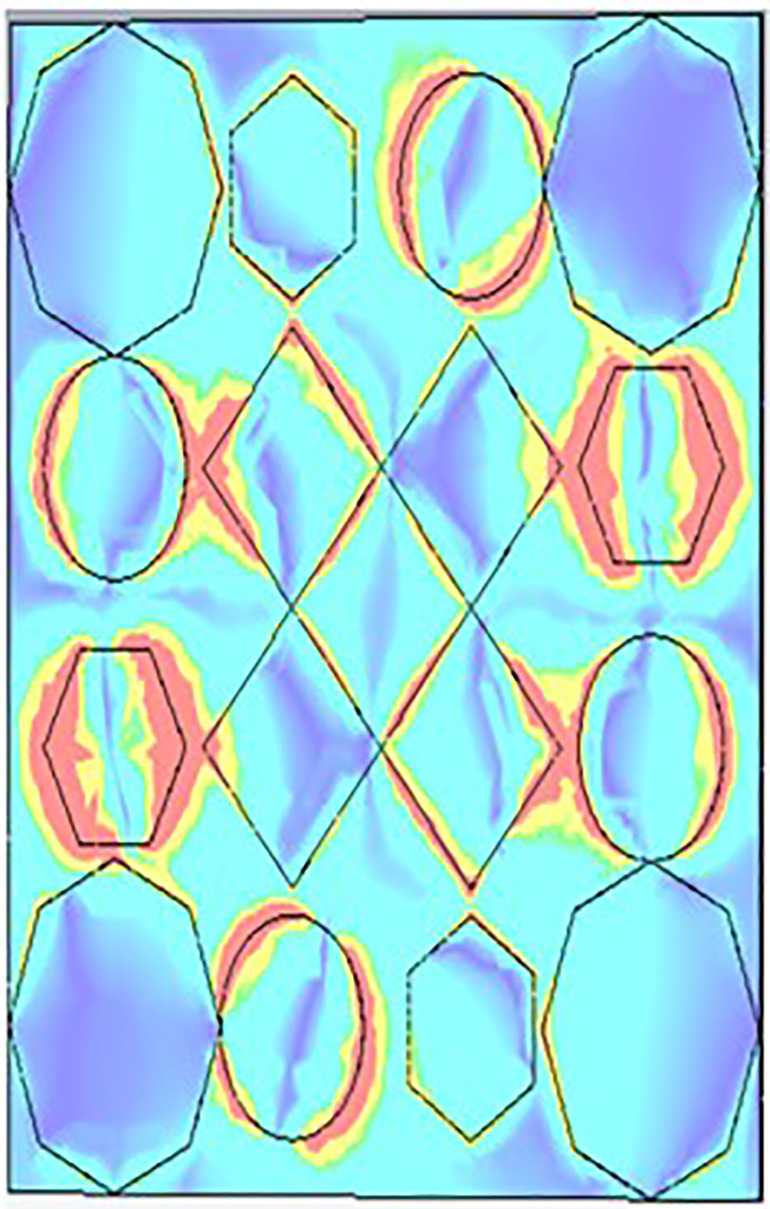
E- field at 10.41GHz.

**Fig 34:**
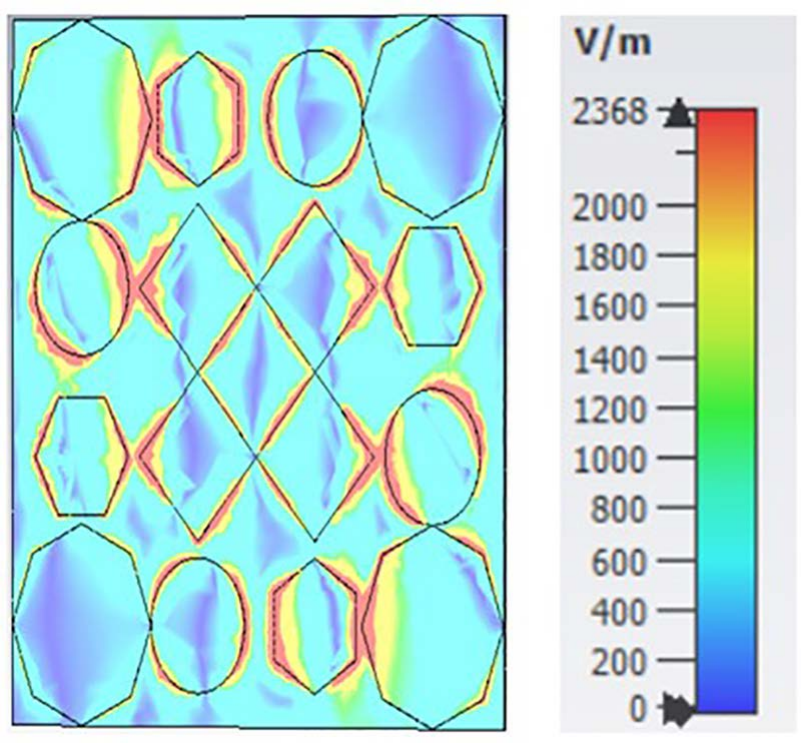
E- field at 11.28GHz.

As shown in Fig 32, 33 and 34, a maximum amount of E-field is formed on the outermost portion of the hexagonal shape.

**Fig 35:**
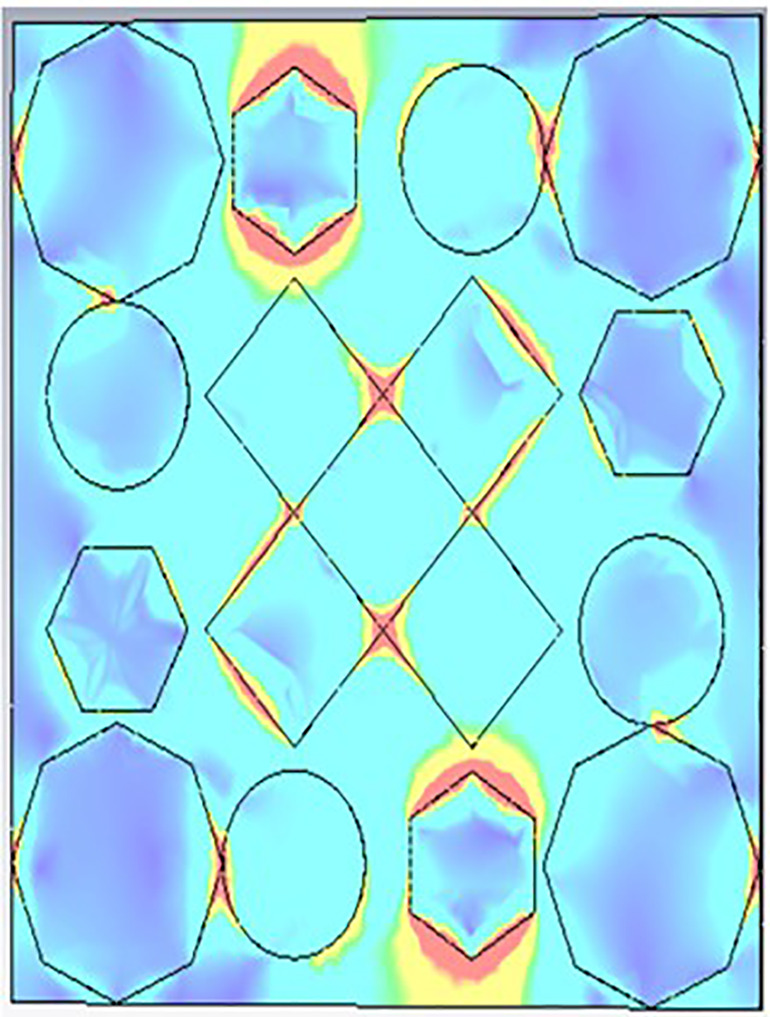
H- field at 9.93GHz.

**Fig 36:**
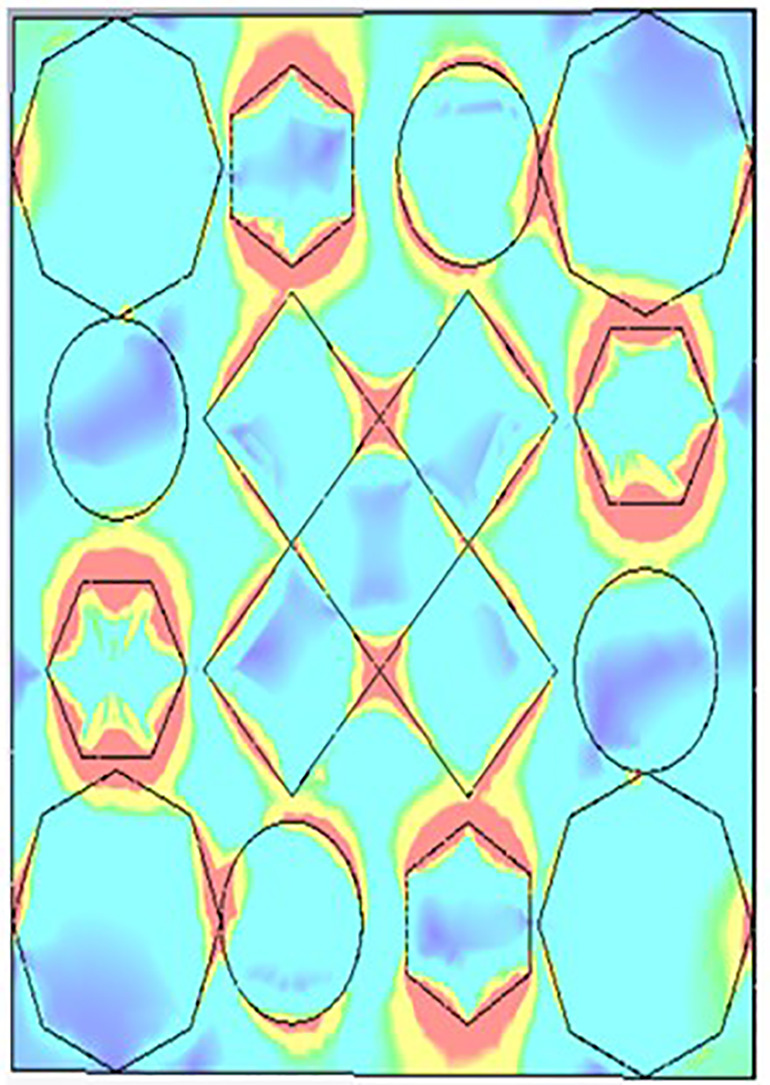
H- field at 10.41GHz.

**Fig 37:**
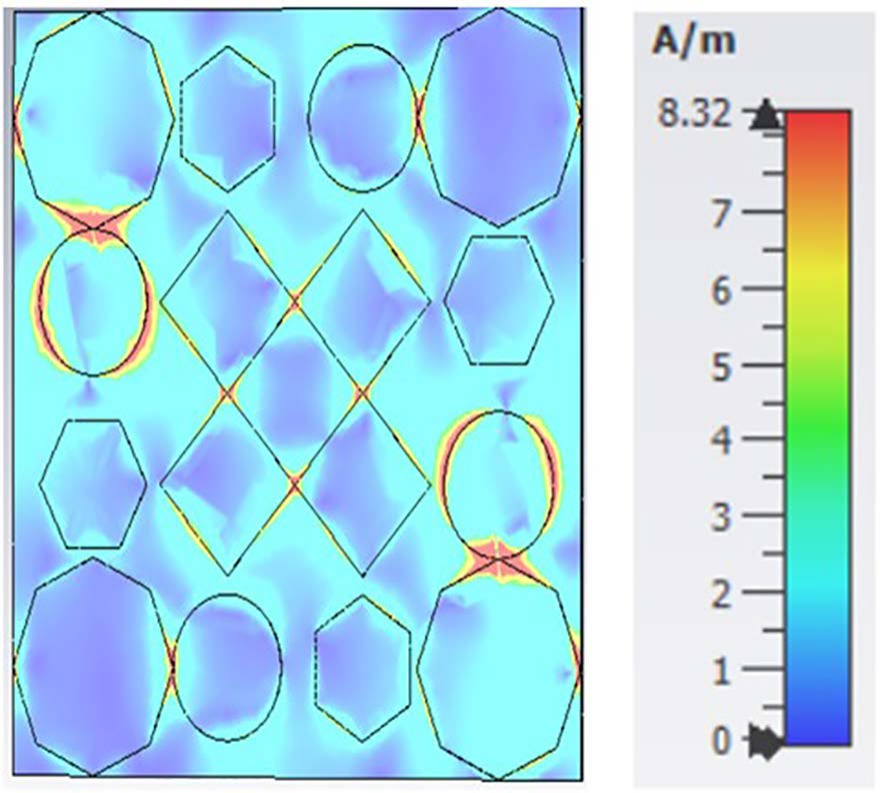
H- field at 11.28GHz.

Fig 35, 36 and 37 show the distributions of the magnetic field (denoted as “H”) at three different frequencies: 9.93 GHz, 10.41 GHz, and 11.28 GHz. The color scale on the right represents the magnetic field strength, where blue indicates lower values and red/yellow indicates higher values in A/m (amperes per meter). The three panels illustrate how the magnetic field intensity changes across a structure that appears to have a periodic arrangement of polygons (hexagons, circles, and diamonds) as the frequency increases.

Each subplot seems to highlight unique patterns in the field distribution, which could be related to the resonant modes or electromagnetic behavior of the structure at different frequencies.

**Fig 38:**
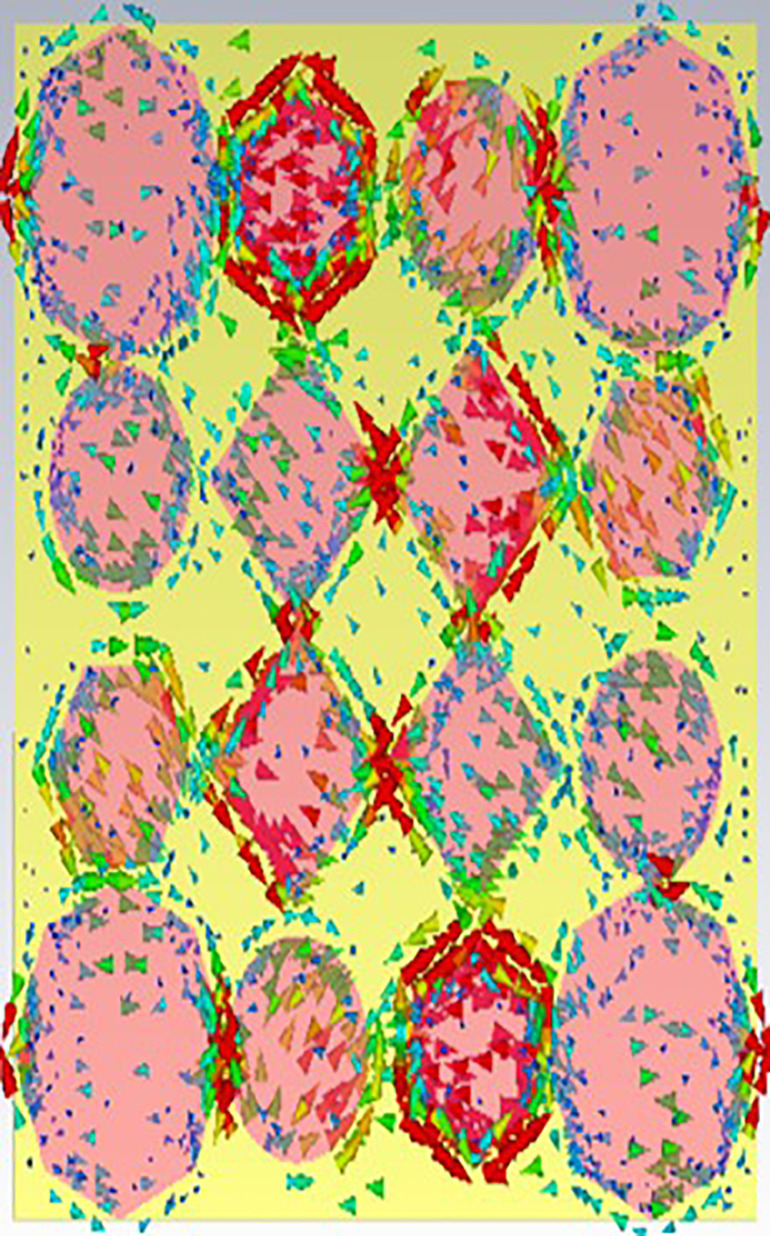
Surface current at 9.93GHz.

**Fig 39:**
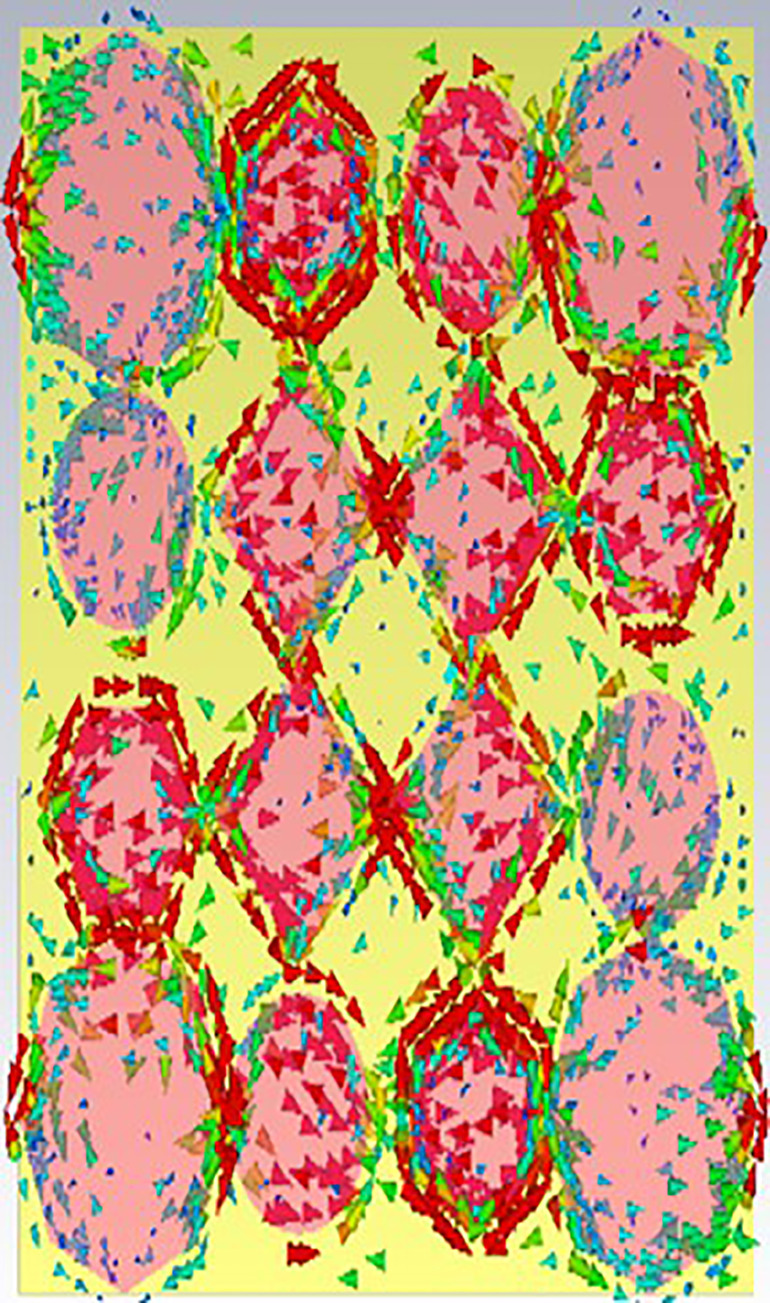
Surface current at 10.41GHz.

**Fig 40:**
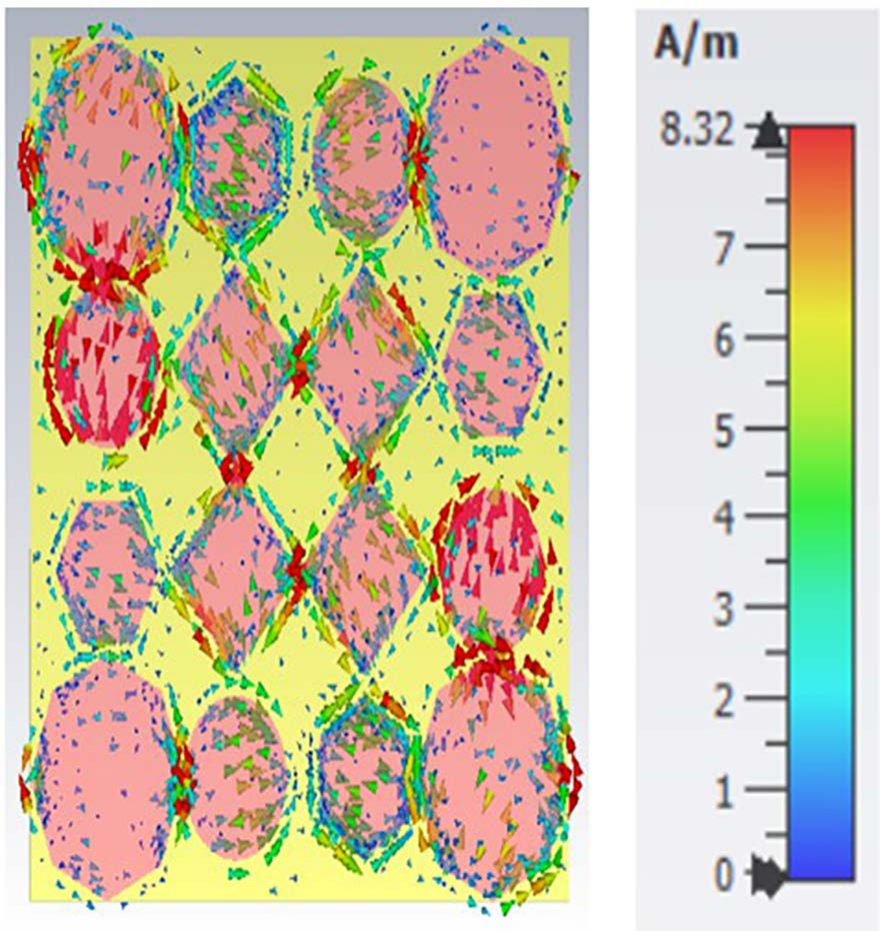
Surface current 11.28 GHz.

Fig 38, 39 and 40 show surface current distributions at three different frequencies: 9.93 GHz, 10.41 GHz, and 11.28 GHz. The color map on the right, in units of A/m (amperes per meter), ranges from blue (low surface current density) to red (high surface current density).

In each of the panels, arrows indicate the direction of the surface current flow. At the same time, the shaded regions, particularly the pink areas, seem to denote zones with higher surface current density. Current distribution varies across polygonal shapes (ovals, hexagons, and diamonds), which likely correspond to specific resonances or modes in the structure. As the frequency increases, the pattern of the surface current appears to change, with certain regions experiencing stronger current flow (denoted by more concentrated red and yellow areas). These shifts in current distribution may indicate the frequency-dependent behaviour of the electromagnetic structure.

The polarization-insensitive behavior of the proposed absorber originates from its geometrically symmetric and quasi-isotropic resonator configuration. Due to the presence of multiple polygonal shapes distributed uniformly within the unit cell, the induced surface currents remain nearly unchanged under rotation of the incident electric field. Consequently, the effective inductive and capacitive responses remain stable for different polarization angles, resulting in consistent absorption characteristics for both TE and TM modes.

## Conclusion

We have produced a new compact and novel non-uniform metamaterial absorber for X-band applications. Several polygon patches in square, octagonal, square, and rhombus forms make up the absorber's proposed unit cell. A parametric study justifies the exact dimensions of the resonator, which are carefully selected with the metamaterials’ properties in mind. The proposed absorber not only boasts a maximum absorption rate exceeding 90%, but also exhibits polarization insensitivity. Furthermore, we go into detail about the effective medium parameters, the frequency characteristics of the E-field, H-field, and surface current of the unit cell. From the obtained results and the left-hand behaviour of the proposed absorber in the X-frequency region, the proposed absorber could be a viable choice for X-band satellite and radar communication applications. The proposed non-uniform polygonal resonator configuration enables three closely spaced absorption peaks with absorptivity exceeding 95%. Owing to its flexibility, high absorption efficiency, and stable performance under real-world conditions, the proposed absorber is a strong candidate for X-band radar, satellite communication and electromagnetic compatibility applications.
